# Comparative hematological and physiological responses to ground and pool training in Colombian Paso Horses

**DOI:** 10.14202/vetworld.2026.1246-1256

**Published:** 2026-03-23

**Authors:** Santiago Lenis-Álvarez, José Ramón Martínez-Aranzales, Maria Patricia Arias-Gutierrez

**Affiliations:** 1 Equine Medicine and Surgery Research Line, CENTAURO Research Group, School of Veterinary Medicine, Faculty of Agricultural Sciences, University of Antioquia, Medellin 050010, Antioquia, Colombia; 2 GINVER Research Group, Faculty of Veterinary Medicine, Corporación Universitaria Remington, Medellin 05000, Antioquia, Colombia; 3 INCA-CES Research Group, Faculty of Veterinary Medicine and Animal Science, CES University, Medellin 050021, Antioquia, Colombia

**Keywords:** aquatic training, Colombian Paso horse, equine physiology, exercise response, hematology, lactate, sport horses, training modalities

## Abstract

**Background and Aim:**

Exercise training causes physiological and hematological changes that are crucial for enhancing athletic performance in horses. While these responses have been extensively studied in various equine breeds, there is limited information regarding Colombian Paso Horses (CPH), especially concerning the comparative effects of different training methods. Ground-based training is frequently used to improve gait and speed, whereas aquatic exercise has become an alternative conditioning approach that lessens musculoskeletal stress. However, the physiological responses related to these training modalities in CPH are not well understood. Therefore, this study aimed to compare the immediate physiological responses of the hemo-leukogram, blood lactate levels, and heart rate in CPH undergoing ground and pool training.

**Materials and Methods:**

Ten clinically healthy CPH horses (6 females and 4 males), with an average age of 10 ± 4 years and a body weight of approximately 350 ± 20 kg, participated in this repeated-measures study. The horses performed high-intensity exercise on ground and aquatic training modalities, with a 30-day interval between protocols. Each session lasted 40 minutes and included warm-up, moderate-to-high-intensity activity, and a cool-down phase. Venous blood samples were collected from the jugular vein before and immediately after exercise to assess hematological parameters and blood lactate levels. Heart rate was continuously monitored using a heart rate monitor. Data were analyzed using either parametric or non-parametric statistical tests, depending on the distribution, with significance set at p < 0.05.

**Results:**

Both training modalities elicited significant post-exercise physiological responses. Ground exercise caused a notable increase in erythrocytes, hematocrit, lymphocytes, and globulins (p < 0.05). Pool training also led to significant increases in erythrocytes, hemoglobin, and hematocrit (p < 0.05). Blood lactate levels rose from approximately 1.04 to 5.80 mmol/L after ground exercise and from 1.29 to 2.46 mmol/L after pool exercise. Heart rate significantly increased in both methods, reaching about 203 bpm during ground exercise and 215 bpm during pool exercise. Long-term adaptations included a significant decrease in resting heart rate after both training protocols, indicating enhanced cardiovascular efficiency. Overall, physiological responses were more pronounced after ground exercise compared to aquatic exercise.

**Conclusion:**

High-intensity ground and aquatic exercises both induce significant hematological and physiological responses in CPH. However, ground-based training causes greater metabolic and hematological changes, indicating higher physiological demands. In contrast, aquatic exercise offers a lower-impact conditioning option that boosts cardiovascular efficiency while reducing mechanical stress. These findings emphasize the potential of integrating aquatic training into conditioning programs for CPH to improve performance and recovery.

## INTRODUCTION

The success of athletic performance relies on training programs that enhance physical fitness and maintain proper body condition [1], while also reducing exercise-induced pathologies [2]. Numerous physiological adaptations to training have been extensively documented in human athletes and sport horses involved in disciplines that require speed, power, and endurance, across different training environments such as hard ground and water-based exercise.

Exercise causes several changes in the hemo-leukogram. These include higher hematocrit and hemoglobin levels due to fluid and plasma loss during physical activity [3]. Changes in total white blood cell count, especially neutrophils and lymphocytes, indicate inflammatory and immune responses linked to exercise-related stress [4]. Increased platelet levels are also common and relate to coagulation and tissue repair after exercise. Additionally, higher blood lactate levels show increased metabolic activity and cellular acidification, which can result in lactic acidosis and contribute to muscle fatigue [5, 6]. These physiological changes may also involve electrolyte imbalances and changes in protein levels [7].

Training protocols and their effects on athletic performance have been extensively described in horses participating in various equestrian disciplines [8]. In Colombian Paso Horses (CPH), high-intensity ground-based training is often used to improve speed and gait execution [9]. Although recent years have seen advances in CPH training and the inclusion of swimming as part of physical conditioning programs, scientific evidence evaluating these training methods remains limited. Recent studies have documented hematological and biochemical responses in obese CPH subjected to field exercise tests [10], as well as changes in metabolic and endocrine profiles following aquatic training programs in horses of the same breed [11].

Despite increasing interest in conditioning strategies for sport horses, scientific understanding of physiological responses to different exercise methods in CPH remains limited. Most existing studies on equine exercise physiology have focused on Thoroughbreds, endurance horses, or other athletic breeds, and their findings cannot be directly applied to CPH because this breed has unique gait mechanics, biomechanics, and training needs. Specifically, CPH are known for highly specialized four-beat gaits and are often trained in field conditions to optimize speed and smoothness. Although ground-based training is the most common conditioning method, aquatic exercise has recently been added to training programs because of its potential to reduce musculoskeletal load while maintaining cardiovascular fitness. However, the physiological responses to aquatic exercise in this breed are still not well documented.

Previous studies have mainly examined isolated physiological indicators or focused on either ground-based or aquatic training separately. Reports on hematological, biochemical, or endocrine responses in CPH remain limited, with most research confined to specific contexts such as field exercise tests or swimming protocols. As a result, there is a lack of controlled studies that simultaneously assess hematological responses, metabolic indicators like blood lactate, and cardiovascular parameters across different training environments. Moreover, the extent of the acute exercise response and the potential long-term adaptations related to these modalities have not been directly compared within the same CPH population. This gap in knowledge hampers the development of evidence-based training strategies tailored to this breed’s physiological traits and limits trainers’ and veterinarians’ ability to optimize conditioning programs while reducing the risk of overtraining or exercise-related disorders.

In light of these limitations, the present study was designed to provide a comparative evaluation of the physiological responses associated with two commonly used training modalities in CPH: ground-based exercise and aquatic exercise. Specifically, the aim of this study was to compare the acute effects of ground and pool training on the hemo-leukogram, blood lactate concentration, and heart rate in CPH subjected to high-intensity exercise. Additionally, the study sought to assess potential training-induced adaptations by analyzing changes in resting physiological parameters following structured training protocols. By integrating hematological, metabolic, and cardiovascular indicators within a repeated-measures experimental design, this research aims to improve understanding of how different exercise environments influence physiological responses in CPH. The findings are expected to contribute to the development of more effective and physiologically appropriate conditioning programs for this breed, supporting improved athletic performance while reducing the risk of excessive physiological stress and musculoskeletal overload.

## MATERIALS AND METHODS

### Ethical approval

This study was approved by the Animal Research Bioethics Committee of the Faculty of Veterinary Medicine, Corporación Universitaria Remington, Colombia, on December 7, 2022, under Act No. 11-2022. Informed consent was obtained from the owners or caretakers of all horses involved in the study.

### Study period and location

The study was conducted from September to December 2024 at a sports training center located in a tropical region at 6º 05’00.84” N latitude and 75º 20’05.23” W longitude, at 2,150 meters above sea level, with a relative humidity of 69%, an average ambient temperature of 16°C, and an annual rainfall of 2,117 mm. The training sessions took place on a longitudinal sand track approximately 40 m long. Aquatic exercises were performed in a circular pool measuring 23.3 m in diameter and 2.9 m deep, with water maintained at around 28°C.

### Study horses

A total of 10 CPH of both sexes (6 females and 4 males), with an approximate weight of 350 ± 20 kg, a body condition score of 5–6/9 [12], and an average age of 10 ± 4 years, were included in this study. All horses were housed in stables and fed a diet consisting of hay (*Digitaria eriantha*), fresh forage (*Pennisetum purpureum*), a commercial concentrate (2 kg/day) (Campeón Dorado®, Solla, Itagüí, Colombia), mineralized salt (60 g/day) (Brío Salts®, Italcol, Girardota, Colombia), and had free access to water.

All animals were clinically healthy and had complete, up-to-date health management plans along with regular dental care. To participate in the study, animals had to be free of orthopedic problems, heart murmurs, arrhythmias, and significant hematological or biochemical abnormalities, and they needed to be actively involved in athletic activities.

### Experimental protocol

After a 30-day acclimation period, during which the horses adapted to the training environments and standardized preparation and exercise protocols, the study was carried out using a repeated-measures design with two exercise methods: ground training and pool training with stationary water flow. The pool water was regularly maintained to ensure proper quality.

Before and after both exercise modalities, horses underwent a general clinical examination that included measuring body temperature (°C), pulse, heart rate, and respiratory rate. Additionally, heart rate (HR)max was recorded using a heart rate monitor during a maximal exercise test on land and a submaximal test in the pool, considering a maximum heart rate stipulated for the breed of 200–220 beats per minute [9].

Once the exercise modality was chosen, all animals completed the respective protocol. In this study, the ground exercise was first performed in a randomly assigned order using the RAND function in Microsoft Excel for Microsoft 365 (Microsoft Corp., Redmond, WA, USA).

Horses underwent a high-intensity training routine in ground work. Each session lasted 40 min and consisted of 10 min of warm-up, 20 min of moderate (70%–80% of HRmax) to high-intensity exercise (80%–90% of HRmax), and 10 min of cool-down walking.

**Figure 1:**
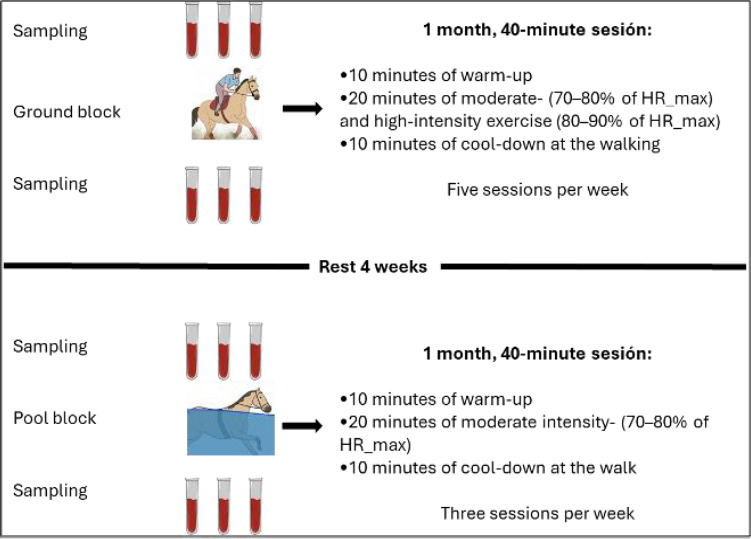
Experimental protocol used for surface-based and pool training modalities in 10 Colombian Paso Horses. Blood samples were collected before and after exercise for each training modality. The rest period and the phases of each exercise session are also described in detail.

In the swimming modality, the horses exercised at moderate intensity (70%–80% of HRmax) in a circular pool with a 23.3-m lap. Each session lasted 40 min, divided into 10 min of warm-up, 20 min of intermittent swimming, and 10 min of stretching exercises followed by a walk to cool-down (Figure 1). During the swimming phase, the horses performed two consecutive laps in one direction, followed by a 1-min pause before switching direction. They swam two laps to the right, paused, and then swam two laps to the left. This cycle was repeated four times, totaling 16 laps in approximately 20 min.

The training frequency was three sessions per week for swimming and five sessions per week for ground training. Finally, this study did not include a control group for any exercise modality; instead, each animal served as its own control and was evaluated before and after each modality.

#### Duration of rest period between modalities

The rest period between both exercise modalities was set at 4 weeks, as recommended by Holcombe *et al*. [13], to prevent potential residual effects on certain variables from the first modality, such as lipopolysaccharide and lactate concentrations, which were studied in a separate project (data not yet published). During this time, the horses engaged in light or low-intensity exercise.

#### Blood sampling

Blood samples were collected before and after the first and last sessions of each training modality, as well as within the first 3 min pre- and post-exercise. Samples were drawn from the jugular vein following a standard preparation and antiseptic protocol prior to venipuncture.

Samples were collected in tubes containing ethylenediaminetetraacetic acid and in tubes without anticoagulant, then transported to the laboratory under refrigerated conditions (4°C) for analysis. Samples with anticoagulant were used for complete blood counts, while samples without anticoagulant were centrifuged at 1,509 × *g* for 10 min, and the resulting serum was used to measure albumin concentration.

#### Hematological analysis

A GENRUI VH-50 automated hematology analyzer (Genrui-bio, Shenzhen, China) was used to measure red blood cell count, hematocrit, hemoglobin levels, white blood cells, neutrophils, lymphocytes, eosinophils, and platelets. Additionally, total plasma proteins were assessed using refractometry.

During hemogram analysis, strict quality control measures were implemented to ensure accuracy and reliability. The hematology analyzer was calibrated using standard solutions and commercial controls, and duplicate samples were analyzed to verify repeatability. Internal controls included reference samples at different levels and manual blood smear reviews to validate leukocyte differentials. For external quality assurance, the laboratory participates in interlaboratory programs and holds ISO accreditation. Routine maintenance and technical checks of the equipment were performed to minimize instrumental bias and ensure consistent performance.

#### Measurement of heart rate and blood lactate

Heart rate was measured using a Suunto Ambit3 Vertical® heart rate monitor (Vantaa, Finland: Suunto; 2021). Lactate concentration was determined with a NOVA Lactate Plus® portable analyzer for quick field assessment (NOVA Biomedical, Waltham, MA, USA), calibrated and validated for equine use.

Lactate was measured using enzymatic-amperometric detection, with lactate oxidase as the reagent in each sensor. No manual calibration was needed because the test strips are pre-calibrated; however, control solutions were used weekly to verify measurement accuracy.

A drop of blood was placed on the device sensor for lactate testing. Measurements were taken while at rest and immediately after exercise (within the first 3 min). The estimated reading time was 10 seconds, and results were given in mmol/L.

### Statistical analysis

The obtained data were stored in a Microsoft Excel® database (Microsoft Corp., Redmond, WA, USA) and then analyzed using SPSS® version 29.0 (IBM Corp., Armonk, NY, USA). A descriptive analysis of all hematological variables, lactate levels, and heart rate was conducted.

Data distribution was evaluated using the Shapiro–Wilk test and Q–Q plots, while homoscedasticity was checked with Levene’s test; when applicable, sphericity was assessed with Mauchly’s test. Variables meeting the normality assumption were expressed as mean ± standard deviation, whereas those with a non-normal distribution were reported as median and interquartile range.

Parametric tests, such as Student’s t-test for both paired and independent samples, were used when the data met normality assumptions. When those assumptions were not met, non-parametric tests were used, specifically the Wilcoxon test for paired data and the Mann–Whitney U test for independent data.

The sample size was based on the total number of individuals available during the study period; therefore, no a priori power calculation was conducted. To compare repeated-measures, linear mixed-effects models were used, with horse included as a random effect. When model assumptions were not satisfied, nonparametric alternatives were employed. Statistical significance was set at p < 0.05.

## RESULTS

### Immediate responses

Immediate physiological responses to exercise were assessed by comparing pre- and post-exercise values at two key points: the first and last day of the training period. Tables 1 and 2 show the average values of hemo-leukogram variables, lactate concentration, and heart rate.

**Table 1:** Comparison of pre- and post-exercise hemo-leukogram variables, lactate concentration, and heart rate in 10 Colombian Paso Horses performing the ground exercise modality.

Parameter	Pre-exercise			Post-exercise			p-value

	Mean ± SD	Min	Max	Mean ± SD	Min	Max	
Erythrocytes (10^6^/mm³)	7.7 ± 0.9	6.2	9.0	11.4 ± 1.0	10.1	12.8	0.001*
Hematocrit (%)	35.4 ± 4.0	29.3	40.1	50.1 ± 6.1	40.1	58.8	0.001*
Hemoglobin (g/dl)	13.3 (10.9–15.2)+	10.1	23.7	16.3 ± 2.2	13.1	21.1	0.123†
Leukocytes (10³/mm³)	10.2 ± 2.3	6.0	14.1	9.8 ± 2.3	6.9	13.7	0.707
Neutrophils (10³/mm³)	4.9 ± 2.8	0.0	9.1	6.4 ± 1.9	3.0	9.4	0.085
Lymphocytes (10³/mm³)	3.6 ± 1.7	0.0	6.1	5.2 ± 1.8	2.9	8.8	0.032*
Eosinophils (10³/mm³)	0.1 (0–0.6)+	0.0	1.1	0 (0–0.1)+	0.0	0.2	0.123†
Platelets (10³/mm³)	240.6 ± 121.51	120.0	451	164.8 ± 48.5	110.0	240	0.132
Total plasma proteins (g/dl)	8.2 ± 2.4	5.9	11.2	7.9 ± 0.8	6.9	8.9	0.652
Albumin (g/dl)	3.4 ± 0.6	2.3	4.1	3.8 ± 0.5	3.1	4.8	0.217
Globulins (g/dl)	3.6 ± 0.6	2.9	4.6	4.1 ± 0.6	3.0	4.8	0.031*
Lactate (mmol/L)	1.04 (0.88–1.37)+	0.58	2.10	5.80 (4.35–11.03)	3.99	12.90	0.005*†
HR (bpm)	89 ± 18	60.0	115	203 ± 10	190	220	0.001*

+: median and interquartile range, †: Wilcoxon test result, SD = standard deviation, HR = heart rate,

* statistically significant (p ≤ 0.05), Student’s t-test.

**Table 2:** Comparison of pre- and post-exercise hemo-leukogram variables, lactate concentration, and heart rate during pool training in 10 Colombian Paso Horses.

Parameter	Pre-exercise			Post-exercise			p-value

	Mean ± SD	Min	Max	Mean ± SD	Min	Max	
Erythrocytes (10^6^/mm³)	8.7 ± 1.7	6.8	11.1	10.4 ± 1.0	8.9	11.9	0.012*
Hematocrit (%)	35.9 ± 4.1	31.0	42.9	46.2 ± 4.5	39.9	53.9	0.002*
Hemoglobin (g/dl)	12.4 ± 1.4	10.9	15.2	16.6 ± 1.4	14.3	18.7	0.001*
Leukocytes (10³/mm³)	7.1 ± 1.4	4.5	8.9	7.0 ± 0.8	5.6	9.0	0.777
Neutrophils (10³/mm³)	3.1 ± 1.1	1.5	5.0	3.6 ± 0.8	2.1	4.7	0.127
Lymphocytes (10³/mm³)	3.1 ± 1.0	1.8	4.9	2.9 ± 1.0	1.9	4.8	0.486
Eosinophils (10³/mm³)	0.03 (0–0.3)+	0.0	0.5	0.3 (0.1–0.1)+	0.1	0.1	0.126†
Platelets (10³/mm³)	227.3 ± 38.30	173	291	217.6 ± 64.5	140	291	0.588
Plasma protein concentration (g/dl)	6.8 ± 0.8	5.6	10.0	7.2 ± 1.3	5.2	10.0	0.465
Albumin (g/dl)	3.4 ± 0.4	2.6	4.1	3.3 ± 0.5	2.4	4.0	0.611
Globulins (g/dl)	3.2 ± 0.4	2.8	4.3	3.2 ± 0.5	2.2	3.9	0.797
Lactate (mmol/L)	1.29 ± 0.98	0.36	3.14	2.46 ± 1.11	0.99	4.10	0.007*
HR (bpm)	94 ± 19	55.0	115	215 ± 7.0	205	225	0.001*

+: median and interquartile range, †: Wilcoxon test result, SD = standard deviation, HR = heart rate,

* statistically significant (p ≤ 0.05), Student’s t-test.

Regarding immediate physiological responses, most pre-exercise values were lower than post-exercise values. In the ground exercise modality, a significant post-exercise increase was observed in erythrocyte count and hematocrit (p = 0.001), as well as in lymphocytes and globulins (p = 0.031). In the pool exercise modality, there was a significant post-exercise increase in erythrocytes (p = 0.012), hemoglobin (p = 0.001), and hematocrit (p = 0.002). Lactate concentration and heart rate followed a similar trend, showing significant post-exercise increases in both modalities (p = 0.001).

### Long-term adaptations

Additionally, post-exercise differences in variables such as erythrocytes, leukocytes, and heart rate were observed between the two exercise modalities (Table 3). A significant decrease in erythrocyte count (p = 0.037), leukocytes (p = 0.002), neutrophils (p = 0.000), eosinophils (p = 0.022), and lymphocytes (p = 0.003) was observed, with these changes being more pronounced in the ground exercise modality. Globulins (p = 0.002) and albumin (p = 0.031) showed significant differences, with higher values in the ground exercise group. Lactate concentration exhibited a notable difference between exercise types, being significantly higher in ground exercise (p = 0.000). Finally, maximum heart rate was significantly lower during swimming.

**Table 3:** Comparison of hematological variables, lactate, and heart rate in 10 Colombian Paso Horses during the post-exercise period between ground and pool exercise modalities (post-exercise ground – post-exercise pool).

Parameter	Mean difference	Confidence interval of the 95% difference		p-value

		Lower	Upper	
Erythrocytes (10^6^/mm³)	1.00	1.20	0.90	0.037*
Hematocrit (%)	3.90	0.20	4.90	0.124
Hemoglobin (g/dl)	−0.30	−1.20	2.40	0.683
Leukocytes (10³/mm³)	2.80	1.30	4.70	0.002*
Neutrophils (10³/mm³)	2.80	0.90	4.70	0.000*
Lymphocytes (10³/mm³)	2.30	1.00	4.00	0.003*
Eosinophils (10³/mm³)	−0.30	−0.10	0.10	0.022*
Platelets (10³/mm³)	−52.80	−30.00	−51.00	0.053
Plasma protein concentration (g/dl)	0.70	1.70	−1.10	0.229
Albumin (g/dl)	0.50	0.70	0.80	0.031*
Globulins (g/dl)	0.90	0.80	0.90	0.002*
Lactate (mmol/L)	3.34	3.00	8.80	0.000*
HR (bpm)	−12.00	−15.00	−5.00	0.010*

## DISCUSSION

### Novelty of the study and overall research context

To our knowledge, no controlled comparison has been made between the immediate and long-term hematological, lactate, and cardiovascular adaptations from ground-based and aquatic exercise in the CPH. This study is the first to combine both acute physiological responses and training-induced changes from two different exercise methods within a standardized training program. In this context, the comparison of high-impact ground exercise and low-gravity aquatic exercise uncovered modality-specific hematological patterns that have not been reported before in the CPH.

### Methodological considerations and study limitations

Although this study contributes to sports medicine in CPH, its results should be viewed with consideration of certain methodological limitations. The small sample size and lack of a control group restrict the ability to draw causal conclusions. However, the repeated-measures design facilitated assessing physiological changes within the same individual over time. Exercise intensity was estimated using heart rate, a practical approach in field conditions but less accurate than direct gas exchange measurements. Additionally, while cortisol was measured as a stress marker, including other physiological indicators could have provided a more detailed understanding of responses to the training environment. These findings highlight the need for future research with larger sample sizes, controlled conditions, and increased statistical power to generalize the results to other populations.

### Exercise-induced hematological responses in CPH

The literature on the immediate effects of ground-based and aquatic exercise in various equine breeds and sport modalities is extensive [14, 15]; however, research on long-term hematological adaptations from structured training programs remains limited [16]. Specifically, information on the CPH is scarce, despite it being considered a high-performance breed due to the physical demands of its activity, which is often conducted under empirical training schemes [8, 9]. This study provides evidence on the immediate physiological changes caused by both ground and aquatic exercise, as well as on long-term hematological adaptations in CPH subjected to a ground-based training program, followed by a four-month rest period and then an aquatic training regimen.

Regarding exercise responses, the hemo-leukogram variables evaluated in this study do not match the patterns reported in previous studies conducted in CPH [10, 11]. However, the results show that hematological changes are affected not only by exercise intensity and duration [17, 18], but also by the type or modality of exercise. Although significant hematological changes were observed in both training modalities, they were more pronounced during ground-based exercise, likely due to higher metabolic demand, which triggered a stronger hematological response.

### Hemoconcentration and erythrocyte responses during exercise

Specifically, a significant increase in red blood cell count and hematocrit was observed, indicating notable hemoconcentration linked to plasma volume loss caused by sweating during exercise. This reflects a combined effect of physical exertion and dehydration. Additionally, these responses may have resulted from fluid redistribution among body compartments and could have been mediated by splanic contraction triggered by rapid adrenergic discharge and increased metabolic demands [2]. In this context, elevated hematocrit is a typical finding during high-intensity exercise because it causes a physiological stress response [10]. Therefore, ground-based exercise seems to demand more physiological effort compared to aquatic exercise, which benefits from buoyancy, hydrostatic pressure, and a lower cardiovascular workload.

### Hemoglobin response and oxygen transport during aquatic exercise

Hemoglobin levels changed significantly between the pre- and post-exercise periods in the pool modality. The increase in hemoglobin during exercise indicates enhanced oxygen transport capacity, which is associated with predominantly aerobic metabolism typical of moderate to low-intensity exercise. This interpretation is supported by heart rate monitoring, which showed moderate intensity during pool exercise, compared to the higher demands observed during ground training. These findings suggest that aquatic exercise produces a less demanding physiological response due to the reduced mechanical load on the musculoskeletal system and the moderating effects of water on fluid loss, body temperature regulation, and consequently hemoconcentration [19, 20].

### Plasma protein responses and metabolic regulation

Plasma protein and albumin concentrations did not show significant changes, despite expectations of a post-exercise increase, especially during ground-based exercise where sweating is more intense, as reported in endurance-tested horses and ground-trained CPH [10, 21]. The type of exercise may have influenced this outcome due to changes in plasma volume, which directly affect these parameters. Moreover, plasma protein concentration is a multifactorial variable influenced by both intrinsic and extrinsic factors, including renal filtration rate, metabolic demand, neuroendocrine regulation, nutritional status, hydration balance, and animal health—factors that contribute to its homeostatic regulation [22].

### Leukocyte dynamics and exercise-induced immune responses

In the hemo-leukogram, greater changes were observed after exercise in the ground modality, shown by a general increase in leukocyte count, although no significant differences were seen in leukocyte subtypes. In contrast, the pool modality did not show notable changes in this parameter. These findings suggest that exercise intensity affects the leukocyte response, as previously described, with muscle fatigue associated with marked leukocytosis driven by neutrophilia [23]. However, as noted in racehorses undergoing repeated training sessions [24], neutrophilia was not seen in this study, nor were fatigue levels enough to indicate a state of exhaustion. Therefore, the neutrophil-to-lymphocyte ratio (10:1), which has been proposed as an indicator of stress, overtraining, or physiological exhaustion in horses [25], was not measured.

The post-exercise increase in lymphocyte count has been extensively studied [26, 27] and is associated with the immune system’s response to physical activity. After exercise, leukocytosis indicates complex immune activation, with possible effects on health and athletic performance [28]. This change was observed only in the ground modality, which involved higher intensity, and, along with the observed rise in globulins, suggests an exercise-induced immune and inflammatory response. This interpretation is further supported by the detection of lipopolysaccharides in the blood of some horses in this group (data not yet published). However, some studies have reported no significant changes in lymphocyte or monocyte counts in horses undergoing intense treadmill exercise [23], indicating that these responses can vary depending on the type, duration, and intensity of exercise, as well as individual factors.

### Variability in hematological responses

Many hemo-leukogram variables in both exercise types showed changes between pre- and post-exercise periods without reaching statistical significance. This may be due to the small number of animals and differences in the intensity and duration of protocols used in previous studies that did demonstrate significant changes [10]. Therefore, further research is needed to develop scientifically grounded training protocols tailored to the specific needs of CPH, as reported in other athletic breeds [29].

### Metabolic and cardiovascular indicators of exercise intensity

Comparison of post-exercise parameters between modalities revealed significant differences in most variables, confirming greater intensity and physiological effort during ground exercise. Meanwhile, other parameters showed no differences in this comparison but did differ from pre-exercise values within each modality, suggesting similar intensity between modalities at this stage, with differences in metabolism and cardiovascular impact, as indicated by increased lactate concentration and heart rate, markers of exercise intensity [1, 30–32]. More recently, the behavior of these two variables has been shown to be useful for estimating aerobic fitness in CPH without speed tests or invasive procedures [33]. These changes were also reported in a recent study comparing treadmills with and without water [34].

### Long-term cardiovascular adaptations to training

Regarding long-term adaptations from the training protocols, this study showed a significant decline in resting heart rate after both programs, indicating enhanced cardiovascular efficiency in horses trained with either method. In the ground training protocol, resting heart rate dropped from 87 bpm to 71 bpm, while in the pool protocol, it decreased from 96 bpm to 83 bpm. This decline aligns with the physiological changes typical of aerobic conditioning, where increased vagal tone and better myocardial function lead to lower basal heart rate, as noted in other horse breeds [35–37].

Although both training protocols were effective, the horses exhibited higher resting heart rates before pool training, possibly because of the 4-month detraining period, which suggests a partial loss of previously acquired cardiovascular adaptations. Nonetheless, their ability to respond to a new training stimulus remained intact, as shown by the significant decrease observed at the end of the second protocol. This underscores the value of aquatic exercise as an effective method for restoring cardiovascular fitness in horses that are either previously trained or detrained, without causing musculoskeletal overload.

### Anticipatory heart rate responses and future research considerations

Moreover, it should be noted that pre-exercise heart rate may be part of the anticipatory response to effort, which is potentially influenced by stress. In this context, the higher pre-exercise heart rate observed in the pool could be linked to the increased alertness caused by water entry, a potentially more stressful stimulus for the horse [2], despite the adaptation period these animals had. However, no heart rate monitoring or electrocardiograms were conducted during swimming, which could have provided additional insights into underwater cardiac function [37]. Additionally, although the shape of the pool may affect exercise development [38], this factor should also be examined in future research.

## CONCLUSION

This study compares physiological responses to ground-based and aquatic exercise in CPH using a standardized training program. Both exercise types caused quick changes in multiple hemo-leukogram variables, along with notable rises in blood lactate and heart rate. Ground-based exercise led to stronger hematological responses, including increases in erythrocyte count, hematocrit, lymphocytes, and globulins, indicating higher metabolic and physiological stress. Conversely, aquatic exercise caused moderate rises in erythrocytes, hemoglobin, and hematocrit, showing a lower physiological challenge due to buoyancy and reduced mechanical stress. Besides these immediate effects, both training methods significantly lowered resting heart rate, pointing to improved cardiovascular efficiency and adaptation to exercise.

From a practical standpoint, these findings emphasize the potential benefits of including aquatic exercise in conditioning programs for CPH. While ground-based training tends to produce stronger hematological and metabolic responses linked to high-intensity performance conditioning, aquatic training provides an effective alternative for maintaining cardiovascular fitness with less musculoskeletal strain. Therefore, swimming-based exercise may be especially beneficial during rehabilitation, recovery, or transitional training periods following detraining.

A key strength of this study is the use of a repeated-measures design, which enabled the assessment of physiological responses within the same animals across two different exercise modalities. This approach minimized variation between individuals and allowed for a direct comparison of acute and training-related adaptations under controlled conditions. Additionally, the integration of hematological, metabolic, and cardiovascular indicators offered a comprehensive evaluation of exercise physiology in CPH, a breed with limited scientific evidence.

However, several limitations should be kept in mind when interpreting the results. The small sample size and lack of an external control group limit the ability to generalize findings or establish clear causal links. Additionally, exercise intensity was estimated through heart rate rather than direct measurements of oxygen consumption or metabolic exchange. Including other physiological markers, such as endocrine or inflammatory indicators, could have provided a more thorough understanding of exercise responses.

Future research should include larger populations of CPH and controlled experimental designs to confirm these findings and expand the understanding of training adaptations in this breed. Studies incorporating advanced physiological monitoring, such as cardiopulmonary assessments, endocrine biomarkers, and biomechanical analyses, would further clarify the mechanisms behind exercise-induced adaptations. Additionally, long-term evaluations comparing different training intensities and aquatic exercise protocols may help develop optimized conditioning strategies for equine athletes.

In conclusion, both ground-based and aquatic exercises are effective conditioning methods for CPH, producing measurable hematological and cardiovascular changes. However, the physiological responses differ between the two, with ground exercise generating greater metabolic demand and aquatic exercise offering a lower-impact option for enhancing cardiovascular efficiency. These findings support the development of evidence-based training strategies aimed at maximizing performance while protecting the health and welfare of equine athletes.

**DATA AVAILABILITY**

The supplementary data can be made available from the corresponding author upon request.

## AUTHORS’ CONTRIBUTIONS

SLA: Designed and conducted the study, collected data, analyzed results, and coordinated the statistical analysis. JRMA: Coordinated and guided the research, conceptualization, and analysis, and drafted and edited the manuscript. MPAG: Developed the study concept and reviewed and edited the manuscript. All authors have read and approved the final version of the manuscript.
